# Ethical tensions in occupational therapy practice: Conflicts and competing allegiances

**DOI:** 10.1177/00084174211021707

**Published:** 2021-10-01

**Authors:** Evelyne Durocher, Elizabeth Anne Kinsella

## Abstract

**Background.:**

Ethical tensions inevitably arise in practice in light of diverse agendas embedded in practice contexts. Such tensions can contribute to moral distress and lead to professional burnout and attrition. Despite potentially serious implications, little work has been done to examine how various allegiances in occupational therapy practice can set up ethical tensions.

**Purpose.:**

In this article, we present findings of an exploratory study examining conflicting allegiances in occupational therapy.

**Method.:**

Using collective case study methodology, we examined ethical tensions reported by seven occupational therapists practicing in different settings in Southwestern Ontario.

**Findings.:**

Ethical tensions were seen to arise in ways that highlighted competing allegiances to participants’ own values, clients, others in the context, colleagues, employers, and regulatory colleges.

**Implications.:**

The findings open a discussion informing how practice settings can better facilitate practice directed at responding to client needs while also meeting the various demands imposed on occupational therapists.

## Abstract (fr)

**Description.:**

Des tensions éthiques se produisent inévitablement, dans la pratique, du fait des divers enjeux prioritaires ancrés dans les contextes de pratique. Ces tensions peuvent contribuer à une détresse morale et mener à l’épuisement professionnel et à l’attrition. En dépit de ces implications potentiellement graves, peu d’études ont examiné dans quelle mesure diverses allégeances peuvent créer des tensions éthiques dans la pratique de l’ergothérapie.

**But.:**

Dans cet article, nous présentons les résultats d’une étude exploratoire des allégeances conflictuelles en ergothérapie.

**Méthodologie.:**

Nous avons utilisé la méthodologie de l’étude de cas multiples pour examiner les tensions éthiques rapportées par sept ergothérapeutes exerçant dans différents contextes du le sud-ouest de l’Ontario.

**Résultats.:**

Les tensions éthiques semblaient se produire lorsque les participants mettaient en évidence des allégeances rivales aux valeurs personnelles des participants, des clients, à d’autres personnes dans le contexte, des collègues, des employeurs et des organismes de réglementation professionnelle.

**Conséquences.:**

Les résultats de cette étude ouvrent une discussion qui éclaire la façon dont les contextes de pratique peuvent mieux faciliter les pratiques centrées sur la réponse aux besoins des clients, tout en répondant aux diverses exigences imposées aux ergothérapeutes.

## Introduction

Ethical tensions have long been recognized as a significant concern in health care practice (Beauchamp & Childress, 2012; Doherty & Purtilo, 2016; Wright-St. Clair & Seedhouse, 2005). Such challenges can contribute to moral distress for practitioners and can have significant personal, professional, and practice implications (Fumis, Amarante, Nasciemento, & Viera, 2017; Pauly, Varcoe, & Storch, 2012; Penny, Ewing, Hamid, Shutt, & Walter, 2014). While some work has examined ethical challenges experienced by health care professionals, little research has specifically explored conflicting allegiances that can contribute to ethical challenges for occupational therapists. In this article, we present a secondary analysis of an exploratory collective case study (Stake, 2000) in which we examine conflicting allegiances that contributed to ethical challenges experienced by occupational therapists.

### Background

#### Ethical tensions in practice

Health care professionals have long experienced ethical (or moral) tensions in practice (Beauchamp & Childress, 2012; Doherty & Purtilo, 2016). Many scholars use the terms ethics and morality interchangeably. Ethics is often considered a more secular term in which “rational and critical reflection” processes are used to inquire into “values, virtues, principles, and norms” (Drolet, 2018, p. 21). Ethics refers to social systems and how values are enacted across groups. Morality is often depicted as individual experiences of the moral realm. With morality, “values and rules of conduct” are informed through “religion, tradition, habits and customs” (Drolet, 2018, p. 22).

Jameton (1984) defined three types of “moral,” or what others have extended to “ethical” (Opacich, 1996; Storch, 2004) tensions: *ethical uncertainty*—uncertainty about whether a situation is an ethical issue or which ethical principles may be relevant; *ethical dilemmas*—situations presenting two or more mutually-exclusive courses of action that clash in values; and *ethical distress*—when a course of action aligning with values and perceived duties is not possible due to internal or external constraints (Jameton, 1984; Bushby, Chan, Druif, Ho, & Kinsella, 2015; Drolet 2018). These situations comprise our use of the term *ethical tensions.* We suggest ethical tensions may be experienced by individuals or collectives, may be shaped by social factors, and may incite ethical reflection and deliberation.

Ethical tensions are prevalent and tangibly shape occupational therapy practice (Drolet, 2018; Drolet, Pinard & Gaudet, 2017; Durocher, Kinsella, McCorquodale, & Phelan, 2016; Kinsella, Park, Appiagyei, Chang, & Chow, 2008; Mackey, 2014; Penny et al., 2014; Wright-St Clair & Seedhouse, 2005). Bushby et al. (2015) identified ethical issues experienced by occupational therapists related to: resource and systemic issues; upholding ethical principles and values; client safety; working with vulnerable clients; interpersonal conflicts; upholding professional standards; and practice management. In Canada, Drolet and Maclure (2016) identified that important values such as patient and professional autonomy, human dignity, occupational engagement, taking a holistic approach, social justice, partnership, and professionalism were often compromised in occupational therapy practice. Further, a Canadian study of students’ experiences pointed to ethical tensions related to systemic constraints, conflicting values, witnessing questionable behaviour, and failure to speak up (Kinsella et al., 2008). Engaging in practices that compromise one’s values is a contributor to ethical tensions and moral distress (Ulrich & Grady, 2018).

#### Negative implications of ethical tensions

The prevalence and increasing complexity of ethical tensions and the moral distress to which these contribute (Kälvemark, Höglund, Hansson, Westerholm, & Arnetz, 2004; Ulrich & Grady, 2018) are alarming, as these have negative implications for health and social care disciplines. Moral distress is defined as “the experience of being seriously compromised as a moral agent in practicing in accordance with accepted professional values and standards” and as “one or more negative self-directed emotions or attitudes that arise in response to one’s perceived involvement in a situation that one perceives as morally undesirable” (Musto & Rodney, 2018, p.12). Fumis et al. (2017) surveyed 283 critical care providers in Brazil; 23% of participants identified experiencing severe burnout linked to moral distress. Canadian nursing researchers linked moral distress to decreased professional satisfaction, recruitment challenges, and difficulty with retention (Pauly et al., 2012). In an American study, occupational therapists reported high levels of moral distress with over half of the 600 participants reporting they had left a position, had considered, or were considering leaving a position due to moral distress linked to their role (Penny et al., 2014). Considering the serious consequences of ethical tensions in practice, professional and regulatory bodies have identified a pressing need to support ethical occupational therapy practice (Drolet, 2018).

### Rationale and Purpose of Article

While there is a growing body of evidence examining contributors to and implications of ethical tensions in occupational therapy, little research explores practice demands that may conflict for occupational therapists. The research presented here is part of an overarching project exploring ethical tensions experienced by occupational therapists in Southwestern Ontario, Canada. Specifically, the purpose of this research is to examine allegiances that may collide in practice, thus contributing to ethical tensions experienced by occupational therapists.

## Method

### Design/Approach

This work builds on a scoping review that identified ethical tensions documented in the occupational therapy literature (Bushby et al., 2015). In our engagement with the literature, we noted that therapists seemed torn in relation to allegiances associated with their role. In this portion of the work, we looked particularly at competing allegiances at the heart of ethical tensions. We define competing allegiances as: values related to perceived professional obligations to stakeholders in practice that call for differing actions, and thus conflict. We explore the research question “What is the nature of tensions reported by therapists, related to competing values and obligations and the consequent actions for which they call?” We employed collective case study methods (Stake, 2000) to explore ethical tensions within occupational therapy. Collective case study methodology enables examination of phenomena in context (in this case, ethical tensions) allowing researchers to explore patterns and differences within and between cases (Stake, 2000). The study was approved by the research ethics board at Western University.

### Participants

Seven practicing occupational therapists participated in the study. Occupational therapy leaders in key organizations across Southwestern Ontario were requested to distribute recruitment notices. Interested therapists contacted the research team. Occupational therapy leaders also suggested interested therapists (with their permission) to the research team who contacted the potential participants.

Stake (2000) calls for variation in cases to maximize learning about the phenomenon. For this reason, purposive sampling (Miles, Huberman, & Saldaña, 2013) was used. Inclusion criteria were: occupational therapists; employed in a hospital or community-based setting; minimum of 2 years experience.

### Data Collection

A semi-structured interview guide was developed, pilot-tested with two individuals, and accordingly refined. The 24 interview guide questions focused on: ethical tensions in practice and how these are experienced and navigated. Examples of questions include: “Have you have experienced ethical tensions in practice? Can you tell me about these experiences and how you negotiated them?”; “Do ethical tensions come to mind in relation to policies or the way things are done in your workplace? Can you tell me about these experiences and how you or others navigated them?”; and “Have you experienced tensions related to: ability/disability, age, gender, race or ethnicity, sexual orientation, other? Can you tell me about these and how you or others negotiated them?” Interviews averaged 90 minutes in length. All interviews were audio-recorded and professionally transcribed; transcripts were reviewed by two researchers to ensure accuracy.

### Analysis

In alignment with collective case study methods (Stake, 2000), the phenomenon of ethical tensions was examined from multiple perspectives. An initial close reading of each transcript was conducted, and a brief report was written about each. The transcripts were analyzed and coded by one researcher using an inductive coding scheme informed by the research purpose. Initial codes focused on the types of ethical tensions and contributing factors that participants reported. Once all transcripts were coded, the codes were organized thematically by the research team. At this point, in alignment with guidance offered by Miles et al. (2013), new codes were identified and others were eliminated or merged. The transcripts were then recoded using the reworked coding scheme. Data were analyzed within and across the cases by two members of the research team who collaboratively categorized ethical tensions as represented below.

Procedural and analytical rigour was enhanced by: (1) clear documentation of processes (Tracy, 2010); (2) a data matrix to display and allow examination of patterns and discrepancies across cases (Miles et al., 2013); and (3) reflexive memoing to help identify implicit assumptions (Green & Thorogood, 2009). Credibility was enhanced through: thick description that included concrete details from the data (Geertz, 1973; Tracy, 2010); collecting data from multiple perspectives; involving individuals with different clinical and research backgrounds in the analysis; and contextualizing data within the literature (Tracy, 2010).

## Findings

The participants’ practice settings included children’s rehabilitation, occupational health and safety, community mental health, forensic mental health, inpatient adult rehabilitation, and private practice. Participants included one man and six women, each with 8–19 years of occupational therapy experience (*M* = 13.4 years).

### Conflicts Between Multiple Allegiances

All participants reported ethical tensions that revealed conflicting allegiances. The findings illuminate tensions between allegiances to: (1) the client: respect for client autonomy and safety concerns; (2) client values and therapist values; (3) colleagues and to clients or regulatory college mandates; (4) therapists’ values and employer directives; and (5) clients and regulatory college mandates (see Table 1).

**Table 1:** Tensions Between Competing Allegiances

	**Allegiance**	**versus**	**Allegiance**
1	Respect for client autonomy	vs	Protecting client safety
2	The client	vs	Therapist’s values
3	Colleagues	vs	Clients or the regulatory college
4	Therapist’s values	vs	Employer directives
5	The client	vs	Regulatory college mandates

#### Tensions between allegiances to the client

Respect for client autonomy and safety concerns. Participants recounted situations of ethical tension related to conflict between upholding client autonomy and maximizing safety of clients or others. Specific examples included situations related to clients’ living circumstances and their use of scooters or cars. Tension between therapists’ desire to uphold clients’ autonomy and an impetus to protect physical safety was frequently noted in situations of discharge planning. Several therapists expressed distress that actions to respect autonomy could place the client or others at risk of harm. For example, Jane reported that respecting a client’s “right” to live at risk caused her anxiety.

“She has the right to make what I felt was potentially the wrong or dangerous decision…because she was making it informed. It was really difficult – I would go home and think about her all night.”

Lynn expressed concern for others’ safety saying:

“I don’t have a problem if a client can make…an informed decision to be unsafe in their home…I do have a problem if they’re living in an apartment building and…I’m afraid they’ll leave the stove on. That has implications…for other people’s safety.”

And

“my spouse will transfer me. My spouse will do this. My spouse will do that. Well, your spouse is frail and elderly too.” (Lynn)

Another example of tension between respect for autonomy and safety was related to reintegration into the community. One participant’s interventions with clients in a forensic mental health unit involved “grading” the return to society. The therapist reported that a client had harmed others but now needed the freedom to go into society to reintegrate. John however noted that caution was required to minimize risks to others and spoke about balancing the client’s autonomy with the safety of others and the clients’ parole limitations:

John: I can figure out how to support this guy to have access…it was graded: 15 minutes, specific geographic area, specific meeting, all the conditions set out in his court order with spot checks.

Interviewer: Essentially to protect the community?

John: Yeah.

John described the tension between helping the client regain the ability to safely be in the community while recognizing he must also consider the safety of the community.

Tensions between autonomy and safety were identified in situations where clients drove mobility devices when it may not be safe for them to do so. Jane discussed such a situation in relation to a motorized scooter. In the initial assessment, she was concerned that the client’s ability to drive the scooter safely was questionable however the scooter mobility greatly increased his quality of life. She therefore provisionally recommended a scooter providing he stays on quieter streets where his performance was stronger. She stated “c*learly he’s not entirely safe to do this but the quality-of-life factor…*” Upon reassessment 6 months later, however, she determined this was no longer safe:

“He’s telling me I can just see your outline. I can’t see your face. When he was writing his consent I had to put the pen exactly where he needed to sign because he couldn’t see…He couldn’t remember where the key went. He couldn’t remember how to adjust the tiller. He told me stories about driving down…that very busy road. He was about 10 blocks past his house – his street - before he realized…I strongly felt he shouldn’t be driving any longer because I didn’t think he was safe and he was potentially putting others at risk too…He was very angry and he accused me of not being supportive and not wanting the best for him…It’s a big sense of loss and he’s already lost a lot and it’s one more thing to lose. I completely empathize with that but he’s not safe. It’s difficult because you want to maintain the independence and their safety at the same time.

Jane was torn between wanting to uphold the client’s autonomy and maximizing his quality of life, however she worried about his safety and that of others. She was additionally concerned about damage to her therapeutic relationship with the client.

In the situations described, participants balanced how much risk they could accept to uphold clients’ autonomy, with consideration of client and community safety.

#### Tensions between allegiances to client and therapist values

Ethical tensions were also identified when clients’ values, personal attributes, or choices did not align with therapists’ values. One such example is discussed above in relation to patient safety. Lynn described another example in which a client had been identified as a paedophile: “*There was a paedophile on the floor…Some people could not treat him because it aroused too many feelings.*” In this case, some practitioners’ values were in tension with their professional mandate to help clients. Connie described an example in which an individual’s wish to be recognized as transgendered was not respected by some professionals because it challenged their values.

Connie: We have a transgendered client…and I feel like it’s unethical how some people on the staff are addressing some of the concerns for that particular client…the client is choosing to wear really tight binding to bind their breasts and one of the therapists in a different discipline is refusing to let that client do physical activity as a coping strategy. Other people are saying it’s self-harm…Staff were concerned about using the client’s preferred name instead of their legal name…whereas for another client using a nickname or an alternative name would just be commonplace.

These scenarios show how individual actions or expressions of gender did not align with values held by some staff members, resulting in clients being treated differently than other clients.

John described the importance of getting past clients’ behaviours despite potential value conflicts:

When you come into work here, you accept that you may work with people who’ve done murders or…sexual assaults or…crimes that are exceedingly horrific to you and your moral compass…. We’re looking at function.

John describes needing to accept that his clients may have harmed others to work to maximize their recovery and growth.

In sum, ethical tensions resulting from conflicts in values or competing allegiances to clients’, colleagues’, or one’s own values were prevalent in the data.

#### Tensions between allegiances to colleagues and to clients or regulatory college mandates

Ethical tensions related to loyalty toward colleagues and a duty to provide the best client care, or to abide by college guidelines included situations in which therapists witnessed colleagues not fulfilling their duties to clients. For example, Jane was called to complete a funding application that a colleague had not finished. The client had already paid the colleague who was not returning the clients’ calls.

I was very torn for several reasons…this therapist should be following through and now the client is going to have to pay me to do the assessment…The therapist had done the script but wouldn’t complete the [funding] application. I really thought to myself whether I should be reporting it to the college.

Jane further discussed that contacting the colleague or doing the job herself entailed further ethical tensions; contacting the colleague would feel like micro-managing; doing the job herself would require a decision to do it pro-bono or to make the client pay her in addition to having paid the colleague. Jane furthermore had to decide whether she would call the college to report this colleague’s behaviour.

Tensions between what participants considered good practice and other team members’ actions were also reported. Lynn discussed a situation in which a physician was making referrals for which he received financial compensation but that she was unsure were warranted.

He gets paid every time he makes a referral…It’s in his best interests to have a lot of referrals when some of them may not be necessary.

Other examples included situations in which only the physician could report a client as an unsafe driver, yet the physician was not doing so:

One physician on the team strongly felt that driving is a right…I felt the exact opposite, that driving is a privilege…It’s a privilege that you need to have adequate skills and safety components to earn…. There were often clients that I felt needed to be reported to the Ministry of Transportation about their ability to safely drive and he would not report them. (Jane)

Witnessing behaviour that participants interpreted as not aligning with codes of conduct created tensions between loyalty to colleagues, with whom participants may have felt it was important to maintain relationships, and commitments to clients, or the professional regulatory college.

#### Tensions between allegiances to therapists’ values and employer directives

Ethical tensions arose in situations in which therapists’ values contrasted with what an employer demanded or expected. In some cases, therapists suggested that what the employer was asking was not in the clients’ best interests or did not align with their regulatory college mandates or personal values. Therapists sometimes stood up for what they believed was right but identified that doing so could create further challenges.

Lynn described a service delivery change that was challenging and not beneficial to clients. Lynn and her colleagues collected data to demonstrate that the change was not meeting the intended aims. The unit manager however was not pleased, and tensions escalated.

Finally I just said, ‘Look, you’re the boss. If you want to veto this, veto it’. Because it was cat and mouse, dancing and challenging, and it became her against me…that became an ongoing tension…things like education requests, people would get funding. I would not…there were consequences.

Lynn described being penalized by her manager for standing up for what she perceived to be in the clients’ and program’s best interests.

In another example, Karen described at times making recommendations that the employer did not support. In such cases, Karen weighed professional risks and potential consequences; sometimes she would stand up for clients and attempt to convince the employer to act differently.

I try to make informed decisions. Sometimes I will get reprimanded. But I always think of what are the consequences and if the consequences are too great to me, for me, I might not have the courage to change it.

Karen further described the arguments she employed to convince her employer to respond to clients’ equipment needs.

We can have that discussion around the cost of a workplace injury and someone being absent from work versus the cost of buying that equipment, because if you look at WSIB, one day lost time is exponentially greater than the cost of a chair or a keyboard tray or other piece of equipment.

Karen, described making an economic argument when the ethical argument was insufficient.

Lynn noted that not all of the conflicting demands set up situations of ethical tension and that some situations arose given the nature of health care practice.

I have an agenda. My focus is the client. But I also understand that managers– they have an agenda too. That’s important as well and I might not know the reasons…Sometimes you have to do things you don’t really want to do or like to do or agree with. But they’re not tensions.

In these examples, participants described situations in which what they perceived as best practice to meet client needs did not align with their employer’s directives. While some of these situations had an ethical aspect, one participant identified that some situations are merely a difference of agenda, which for her did not create an ethical tension.

#### Tensions between allegiances to clients and regulatory college mandates

Participants frequently reported situations in which what they thought was best practice in terms of building and maintaining therapeutic relationships or ensuring that clients received optimal services came into tension with regulatory college guidelines.

Situations exemplifying such tensions included circumstances in which clients wished to express gratitude by giving the therapist a gift or buying them a coffee. Several participants noted that they did not wish to offend clients by refusing small gifts, and that refusing a gift could damage a therapeutic relationship. Fiona discussed this tension saying:

How do you tell a proud man that you have worked and built this therapeutic rapport with, he can’t buy you a $1.25 coffee? How rude is that? It’s a slap in the face because then it breaks that relationship that you’ve built.

Fiona feared damaging the therapeutic relationship by not accepting the gesture of buying a coffee. Similarly, Laura described a situation encountered during a holiday period:

Accepting a gift from the client…it’s definitely something that tears at me. Because of the long-term working relationships…[Clients] don’t understand; they’re like ‘it seems a silly rule…I’ve known you for three years. I want to say thank you. Here’s a small token. I’ve bought’…at Christmas, you can see that clients have a stack of cards and a stack of boxes of chocolates and everybody on their treatment team is getting this appreciation. Not all colleges are as strict so for other people to be accepting it and then I’m not…Many [clients] feel – especially from a cultural perspective - that I’m being disrespectful…I struggle.

The issue was further complicated in situations where the participants would no longer be seeing the client and returning the gift would involve travelling a large distance or shipping fees. Connie summed this up: “*you get to the point where returning the gift is just going to cause more problems.”*

While some therapists reported understanding the reasoning behind the guidelines, gift-giving was frequently noted to create ethical tensions between following regulatory guidelines and social conventions in the interest of maintaining therapeutic relationships.

Therapists knowing clients outside of practice created other situations in which regulatory college guidelines recommended practice that some participants suggested created ethical tensions. Laura expressed the desire to provide care to people she knows but noted this could be perceived as a conflict of interest.

I’m torn…We’re in this profession because we want to help people. I want to help them but I don’t want to put them in a position where it can – down the road - be a detriment.

Laura further argued that she recognizes that in some roles there might be more freedom to show favouritism to certain clients, but that in her role in the publicly-funded health care system, she is limited.

What kind of favouritism could I show? What am I going to do that’s super extra special for you that I wouldn’t do for another client?…In an auto insurance world, the outcomes can be much bigger.

Laura asserts that it could harm the client in the long run if it were perceived that she showed favouritism because she knew the client.

Karen discussed the issue of knowing clients outside of the workplace, which at times can blur the lines between social visits and therapeutic interactions.

I’ve seen some clients multiple times over the years so while it is still a therapeutic relationship, we know each other more on a personal level. They know about my kids and sometimes it comes into more of that social part…this [town] is…relatively small – you do see people…outside of work.

Karen elaborated that the social aspect of the relationship can get in the way of therapy, saying she has to steer the discussion “*back to what we have to do”*, especially with some clients who *“really want to know stuff about the kids [or] really want me to bring in pictures*.” Knowing clients outside of work was recognized as potentially presenting a conflict of interest.

Another example in which ethical tensions related to regulatory guidelines arose involved clients’ choices of vendors. College guidelines stipulate that therapists must show neutrality about vendors. Therapists noted that tensions sometimes arose if the number of available vendors was limited, the therapist’s experience is that one vendor is less responsive or skilled, or a vendor is promoted by an employer organization. Fiona discussed an example prescribing wheelchairs:

We only had two vendors. One that was superior…Amazing!…there was another vendor who…did the bits and bites and the pieces and the wheels…but not the seating principles, not the occupation, not the foundational things…When parents say…and I mean that’s an ethical thing–“Which one do you think? I only want the best for my child”…I would say go visit each one of them…I knew once they went to this one place that they would feel over the moon about it. But because of the big name of this one vendor…people felt obligated almost to go with them…How can I be recommending service that I know is…way below, I would never want my kid to go there.

These situations—gift giving, relationships beyond work, knowledge about vendor quality, etc.—created ethical tensions in which participants identified a conflict between what they perceived to be in clients’ best interests, and practice mandated by the regulatory college. In the situations described above, participants described ethical tensions related to different allegiances that can have significant implications for practice.

## Discussion

This study highlights complexities related to perceived professional practice obligations to stakeholders, which may call for differing and potentially conflicting actions. Occupational therapists hold allegiances that can conflict and compete, setting up situations of ethical tension.

Conflicting allegiances for occupational therapists in this study included allegiances to clients, which involved wanting to meet client needs and maximize well-being as much as possible; to professional colleges, which regulate the profession through specific practice guidelines; to employers and the services in which participants work, which generally have stated aims for processes and practice outcomes; to colleagues, with whom the participants will continue to work; to individuals in client contexts such as family members or caregivers; and finally, to the health care system and society as a whole, whose resources are shared between all individuals who are affected by, or contained in, the context of this health care system. Tensions between multiple and often competing allegiances resulted in situations in which participants were challenged in their attempts to meet the needs or aims of different stakeholders; it was not always clear which or whose aims/agenda/needs should take precedence. Participants described feeling compelled to act in ways that challenged their values, created tensions with regulatory guidelines, may not align with what they felt was in clients’ best interests, strained relationships with colleagues, or potentially put individuals (clients or others in the context) at risk.

The results of this study reflect findings of previous work exploring ethical tensions in occupational therapy practice. The first theme reflects tensions between upholding clients’ autonomy and concern for their safety, and the second one reflects tensions between client and therapist values. Similarly, in a scoping study exploring ethical tensions in occupational therapy practice, Bushby et al. (2015), identified challenges in upholding various ethical principles including client autonomy, particularly when doing so might place the client at risk of harm. Similarly, two values that Drolet and Maclure (2016) identified as being compromised in occupational therapy practice were respect for patient autonomy and respect for patient dignity, which they found conflicted at times with patient safety, or with therapists’, employer, or organizational values. In addition, these first two themes align with the results of a study exploring ethical tensions in private occupational therapy practice that reported tension between upholding clients’ autonomy and continuation of driving, and protecting client or public safety (Goulet & Drolet, 2017). The results related to tensions between allegiances to colleagues, clients, and the regulatory college echo findings in other studies such as themes of interpersonal conflicts and practice management identified by Bushby et al. (2015), discussions of professional autonomy discussed by Drolet and Maclure (2016), challenges in competing allegiances between duties to patients and third-party payers or employers described by Goulet and Drolet (2017), and the theme of being professional identified by Kassberg and Skar (2008) in their study exploring ethical tensions in occupational therapy practice in Sweden. Finally, some of the identified tensions between respect for client and therapist values, and between allegiances to therapists’ values and employer directives aligned with findings by Kinsella et al. (2008) in a study exploring ethical tensions experienced and witnessed by occupational therapy students, as well as in the works of Kassberg and Skar (2008) and Drolet and Maclure (2016).

In contemporary practice contexts, occupational therapists work to meet clients’ needs, but also aim to meet employers’ goals and work within constraints of their role or context while fulfilling obligations to regulatory colleges. At times, allegiances to different stakeholders and/or perceived professional obligations can conflict and therapists may feel compelled to act in ways that compromise values, leading to ethical tensions. An important point outlined in the results is the question about whether the issue is ethical or whether it is merely a challenging situation related to personal conflict, difficult employment circumstances, or complex role demands. Certainly, countless circumstances related to role or employment demands can be difficult; why then would it be relevant whether the challenges are ethical in nature? While non-ethical struggles can contribute to myriad negative circumstances, ethical tensions have been linked to moral distress (Kälvemark et al., 2004; Ulrich & Grady, 2018), which, as discussed above, has been associated with a low sense of accomplishment, decreases in professional satisfaction (Ando & Kawano, 2018; Pauly et al., 2012), professional burnout, attrition (Fumis et al., 2017; Penny et al., 2014; Ulrich & Grady, 2018), and occupational alienation (Durocher et al., 2016). All these consequences can have significant implications for the wellbeing of practicing occupational therapists in their role, as well as more broadly on their career and life trajectories should they make decisions based on ethical tensions and consequent distress experienced in their roles. There could subsequently be significant reverberations for employers who may have to deal with increased sick or stress leave, or with a higher employee turnover. Clients may experience higher turnover of occupational therapists, requiring them to start again with new therapists more frequently, or may not get as efficient service should the therapists be suffering from moral distress.

Given the potentially severe repercussions of ethical tensions for multiple stakeholders, it is important to recognize, document, and engage a profession-wide dialogue about the nature of such tensions in occupational therapy practice. Doing so would enable exploration of how situations of competing allegiances could be minimized through changes in roles and workplace structures to provide more supportive and enabling workplaces that promote a greater recognition for therapists’ professional autonomy. It is furthermore imperative to equip occupational therapists with capabilities to identify, discuss and navigate circumstances that have ethical overtones (Canadian Association of Occupational Therapists, 2016; Drolet, 2018). Simply knowing that many others face ethical tensions may assist therapists in feeling less isolated. Further, recognizing that regulatory colleges are guided by judgments that therapists in similar situations might reasonably make can assist therapist in their deliberations.

Equipping occupational therapists with the capabilities to identify and navigate ethically challenging tensions must begin in the foundational stages of their career. Providing education in preservice occupational therapy programs about ethics, ethical implications in practice and moral distress is imperative in guiding student occupational therapists to develop knowledge to inform ethical practice and mitigate the toll of moral distress that accompanies the ethical tensions they will inevitably face. Perhaps it is time for occupational therapy accreditation bodies to evaluate the breadth and depth of ethics education within professional curricula, and review and mandate ethics education as part of the accreditation process.

For practicing clinicians, a variety of guides and practical approaches may assist therapists to discern whether a situation has ethical implications or not, and to consider how to act. For example, provincial regulatory colleges and national associations have published codes of ethics containing information about ethical and professional values relevant to practice (see Canadian Association of Occupational Therapists, 2016; College of Occupational Therapists of Manitoba, 2010). Ethical issues in practice however are complex and information offered in codes of ethics is insufficient in guiding clinicians to navigate ethical tensions. Kinsella (2012) has identified the need to support practitioners in the development of capacities for reflection, dialogue, professional judgement, and discernment of wise action in the face of morally complex practices. Drolet (2018) has developed an ethical reflection and deliberation framework that may be useful to therapists in identifying and navigating ethical tensions in practice. Another tool is the Conscious Decision-Making Process proposed by the College of Occupational Therapists of Ontario (2012), which guides therapists in reflective decision-making. Guided processes of reflection will not *eliminate* ethical tensions but can allow for informed decision-making and professional judgements that guide actions. The discipline of occupational therapy might consider following the lead of the legal profession which in Ontario mandates a minimum of 12 h per year of Continuing Professional Education, with 3 h needing to be accredited by the Law Society of Ontario (2019) in ethics-related areas.

Codes of ethics and other tools or techniques to guide decision-making and reflection may help to reduce moral distress but are unlikely to eliminate it. Diverse approaches to address moral distress have been identified and may be fruitful to occupational therapy. Beumer (2008) found that workshops about moral distress and coping skills significantly improved intensive care nurses’ perceptions of whether they had the resources to deal with morally distressing situations. One health care institution implemented moral distress consultation services as a successful institutional intervention (Hamric & Epstein, 2017). Other suggested interventions include early identification and recognition of moral distress; providing support and empowerment for individuals to speak up in difficult situations; promoting resolutions that may better align with individuals’ moral compasses; promoting engagement in one’s workplace; and organizational changes that could reduce the incidence of situations that contribute to moral distress (Bong, 2019; Hamrick & Epstein, 2017; Pavlish, Bown-Saltzman, So, & Wong, 2016).

Recognition of how competing allegiances can create ethical tension and moral distress is a matter of attention at many levels. Interventions to change workplace conditions that contribute to moral distress, as well as approaches that assist therapists in developing capacities for reflective judgement and resilience are warranted.

### Future Research

Much of the research about ethical tensions and moral distress has been conducted in the field of nursing. While some findings may transfer to occupational therapy, there is an ongoing need for specific research about conflicting and value-laden allegiances that therapists may encounter, consequent ethical tensions that may arise, and how therapists can mitigate such tensions. Studies that illuminate ways of navigating ethical tensions and moral distress would glean important insights to support occupational therapists in professional practice.

### Limitations

The study is limited by a small sample of participants, in one geographic area, and by the exploratory nature of the work. The results may hold resonance or be practically transferable to other settings, however, they are not generalizable. A limitation may be that the design did not specifically set out to examine competing allegiances; further studies that focus more particularly on this phenomenon, or that concentrate on particular practice areas would be interesting as next steps. Another limitation is that ethical tensions are complex and nuanced and can be difficult to discuss, as such follow-up interviews may have permitted participants to further discuss nuances related to ethical tensions experienced in practice.

## Conclusion

In increasingly complex health and social care practice contexts, occupational therapists are committed to meeting clients’ needs and to do so in alignment with, and within the constraints of, their role, employment context, and regulatory college guidelines. In this study, occupational therapists identified ethical tension frequently arose in relation to competing demands between allegiances to clients, colleagues, employers, regulatory colleges, and their own values and principles. Such tensions may place therapists in situations whereby no apparent option will meet all demands and ethical values will be compromised. Such scenarios could result in moral distress, which can have nefarious implications for occupational therapists and health and social care settings. Bringing to light the potential for complex and competing demands inherent in an occupational therapist’s role can spur discussion about systemic changes that could reduce the incidence of such conflicts and promote the identification and generation of resources and approaches to help occupational therapists facing such conflicts. A variety of approaches have been proposed to promote reflective decision-making and capacities for professional judgement. Further, education and institutional interventions to address moral distress are highlighted recognizing that little research in this realm is focused on occupational therapists.

## Key Messages

- Occupational therapists frequently experience ethical tensions related to conflict between allegiances to multiple stakeholders.
- Conflicts between competing demands can contribute to ethical tensions or can be mere workplace challenges.
- More research is required to identify approaches to help occupational therapists to navigate ethical tensions and related moral distress.

## References

[bibr1-00084174211021707] Ando M. Kawano M. (2018). Relationships among moral distress, sense of coherence, and job satisfaction. Nursing Ethics, 25(5), 571–579. 27531818 10.1177/0969733016660882

[bibr2-00084174211021707] Beauchamp T. Childress J. (2012). Principles of biomedical ethics (6th ed.). Oxford, UK: Oxford University Press.

[bibr3-00084174211021707] Beumer C. (2008). Innovative solutions: The effect of a workshop on reducing the experience of moral distress in an intensive care unit setting. Critical Care Nursing, 27(6), 263–267. 10.1097/01.DCC.0000338871.77658.03 18953194

[bibr4-00084174211021707] Bong H. (2019). Understanding moral distress: How to decrease turnover rates of new graduate pediatric nurses. Pediatric Nursing, 45(3), 109–114.

[bibr5-00084174211021707] Bushby K. Chan J. Druif S. Ho K. Kinsella E. A. (2015). Ethical tensions in occupational therapy practice: A scoping review. British Journal of Occupational Therapy, 78(4), 212–221.

[bibr6-00084174211021707] Canadian Association of Occupational Therapists. (2016). Code of ethics. Retrieved from https://www.caot.ca/site/pt/codeofethics?nav=sidebar

[bibr7-00084174211021707] College of Occupational Therapists of Manitoba. (2010). Code of ethics. Retrieved from https://cotm.ca/upload/COE_2010.pdf

[bibr8-00084174211021707] College of Occupational Therapists of Ontario. (2012). Conscious decision-making in occupational therapy practice. Retrieved from https://www.coto.org/docs/default-source/default-document-library/conscious_decision_making.pdf

[bibr9-00084174211021707] Doherty R. Purtilo R. (2016). Ethical dimensions in the health professions (6th ed.). St Louis, MI: Elsevier.

[bibr10-00084174211021707] Drolet M. J. Maclure J. (2016). Les enjeux éthiques de la pratique de l’ergothérapie: Perceptions d’ergothérapeutes. Approches inductives en pédagogie, 3(2), 166–196.

[bibr11-00084174211021707] Drolet M. J. (2018). Acting ethically. Ottawa, ON: Canadian Association of Occupational Therapists.

[bibr12-00084174211021707] Drolet M. J. Pinard C. Gaudet R. (2017). Les enjeux éthiques de la pratique privée: des ergothérapeutes du Québec lancent un cri d’alarme. Ethica, 21(2), 173–209.

[bibr13-00084174211021707] Durocher E. Kinsella E. A. McCorquodale L. Phelan S. (2016). Ethical tensions related to systemic constraints: Occupational alienation in occupational therapy practice. OTJR: Occupation, Participation & Health, 36(4), 216–226. 10.1177/1539449216665117 27591435

[bibr14-00084174211021707] Fumis R. Amarante G. Nasciemento A. Viera J. (2017). Moral distress and its contribution to the development of burnout syndrome among critical care providers. Annals of Intensive Care, 7(71), 1–8. 28639161 10.1186/s13613-017-0293-2 PMC5479870

[bibr15-00084174211021707] Geertz C. (1973). The interpretation of cultures: Selected essays. New York, NY: Basic Books.

[bibr16-00084174211021707] Goulet M. Drolet M. J. (2017). Les enjeux éthiques de la pratique privée de l’ergothérapie: perceptions d’ergothérapeutes. Bioéthique Online, 6(6), 1–8.

[bibr17-00084174211021707] Green J. Thorogood N. (2009). Qualitative methods for health research (2nd ed.). Thousand Oaks, CA: Sage Publications.

[bibr18-00084174211021707] Hamric A. Epstein E. (2017). A health system-wide moral distress consultation service: Development and evaluation. HEC Forum, 29(2), 127–143. 28070806 10.1007/s10730-016-9315-y

[bibr19-00084174211021707] Jameton A. (1984) Nursing Practice: The Ethical Issues. Englewood Cliffs, NJ: Prentice-Hall.

[bibr190-00084174211021707] Kälvemark S. Höglund A. Hansson M. Westerholm P. Arnetz B. (2004). Living with conflicts-ethical dilemmas and moral distress in the health care system. Social Science and Medicine, 58(6), 1075–1084. 14723903 10.1016/s0277-9536(03)00279-x

[bibr20-00084174211021707] Kassberg A. Skar L. (2008). Experiences of ethical dilemmas in rehabilitation: Swedish occupational therapists’ perspectives. Scandinavian Journal of Occupational Therapy, 15(4), 204–211. 18609243 10.1080/11038120802087618

[bibr21-00084174211021707] Kinsella E. A. (2012). Practitioner reflection and judgement as phronesis. In Phronesis as professional knowledge: Practical wisdom in the professions. Rotterdam: Sense.

[bibr22-00084174211021707] Kinsella E. A. Park A. Appiagyei J. Chang E. Chow D. (2008). Through the eyes of students: Ethical tensions in occupational therapy practice. Canadian Journal of Occupational Therapy, 75(3), 176–183. 10.1177/000841740807500309 18615929

[bibr23-00084174211021707] Law Society of Ontario. (2019). Accreditation criteria. Retrieved from https://lso.ca/lawyers/enhancing-competence/cpd-accreditation-for-licensees/accreditation-criteria.

[bibr24-00084174211021707] Mackey H. (2014). Living tensions: Reconstructing notions of professionalism in occupational therapy. Australian Occupational Therapy Journal, 61(3), 168–176. 24325329 10.1111/1440-1630.12097

[bibr25-00084174211021707] Miles M. Huberman M. Saldaña J. (2013). Qualitative data analysis (3rd ed.). Thousand Oaks, CA: Sage Publications.

[bibr26-00084174211021707] Musto L. Rodney P. (2018). What we know about moral distress. In Ulrich C. Grady C. (Eds.), Moral distress in the health professions (pp. 9–20). Switzerland: Springer.

[bibr260-00084174211021707] Opacich K. (1996). Ethical dimensions in occupational therapy. In The occupational therapy manager. (p. 627–661). Bethesda, MD: AOTA.

[bibr27-00084174211021707] Pauly B. M Varcoe C. Storch J. (2012). Framing the issues: Moral distress in health care. HEC Forum, 24 (1), 1–11. 22446885 10.1007/s10730-012-9176-y PMC3348467

[bibr28-00084174211021707] Pavlish C. Bown-Saltzman K. So L. Wong J. (2016). An evidence-based model for learners addressing moral distress. Journal of Nursing Administration, 46(6), 313–320. 10.1097/NNA.0000000000000351 27214334

[bibr29-00084174211021707] Penny N. Ewing T. Hamid R. Shutt K. Walter A. (2014). An investigation of moral distress experienced by occupational therapists. Occupational Therapy in Health Care, 28(4), 382–393. 25050893 10.3109/07380577.2014.933380

[bibr30-00084174211021707] Stake R. E. (2000). Case studies. In Denzin N. K. Lincoln Y. S. (Eds.), Handbook of qualitative research (2nd ed.) (pp. 435–454). Thousand Oaks, CA: Sage Publications.

[bibr310-00084174211021707] Storch J. (2004). Nursing ethics: A developing moral terrain, In Storch J. Rodney P. Starzomski Rosalie (Eds.) Toward a moral horizon. (pp. 1–16). Toronto, ON: Pearson Education.

[bibr31-00084174211021707] Tracy S. (2010). Qualitative quality: Eight “Big-tent” criteria for excellent qualitative research. Qualitative Inquiry, 16(10), 837–851.

[bibr32-00084174211021707] Ulrich C. Grady C. (2018). Moral distress in the health professions. Switzerland: Springer.

[bibr33-00084174211021707] Wright-St Clair V. Seedhouse D. (2005). The moral context of practice and professional relationships. In Whiteford G. Wright-St Clair V. (Eds.), Occupation and practice in context (pp.16–33). Marrickville, NSW: Elsevier.

